# E-Nose Discrimination of Almond Oils Extracted from Roasted Kernels

**DOI:** 10.3390/nu15010130

**Published:** 2022-12-27

**Authors:** Manuel Álvarez-Ortí, José Emilio Pardo, Gema Cascos, Ramiro Sánchez, Jesús Lozano, Daniel Martín-Vertedor

**Affiliations:** 1 Higher Technical School of Agronomy and Forestry Engineering, University of Castilla-La Mancha, Campus Universitario, s/n, 02071 Albacete, Spain; 2 Technological Institute of Food and Agriculture CICYTEX-INTAEX, Junta of Extremadura, Avda, Adolfo Suárez, s/n, 06007 Badajoz, Spain; 3 Industrial Engineering School, University of Extremadura, Campus Universitario, Av. de Elvas, s/n, 06006 Badajoz, Spain

**Keywords:** sensory quality, thermal treatment, volatile compounds, electronic nose

## Abstract

Almonds contain around 50% fat with a health-promoting fatty acid profile that can be extracted by pressing to obtain high-quality oils. To improve oil sensory properties, the almonds can be subjected to roasting treatments before oil extraction. However, intense thermal treatments may cause the appearance of undesirable volatile compounds causing unpleasant aromas. Thus, oils from almonds subjected to different roasting treatments (30, 45, 60 and 90 min at 150 °C) were analyzed from sensory and the chemical points of view. In addition, an electronic device (E-nose) was used in order to evaluate its usefulness in discriminating samples according to their aromas. The almonds’ roasting treatments caused changes in the sensory properties, since defects such as a burned, dry smell or wood fragrance appeared when almonds were subjected to roasting treatments (>45 min). These data agree with the analysis of volatile compounds, which showed an increase in the content of aldehyde and aromatic groups in roasted almonds oils while alcohols and terpenes decreased. Partial least squares discriminant analysis and partial least squares obtained from the E-nose were able to classify samples (97.5% success) and quantify the burned defect of the oils ($R_{p}^{2}$ of 0.88), showing that the E-nose can be an effective tool for classifying oils.

## 1. Introduction

The almond is the most consumed nut worldwide; an increase in consumption in the last decades has caused an increase in production to more than four million metric tons in 2020 [1]. Almond consumption is on the rise since it is considered a nutrient-dense food according to its content of proteins, carbohydrates, fiber and other macro- and micro-minerals [2,3,4]. In addition, almonds contain about 50% fat, with a healthy fatty acid pattern where oleic acid (about 70%) and linoleic acid (about 20%) predominate and a low percentage of saturated fatty acids (less than 10%) [5]. Moreover, it also contains other minor compounds with antioxidant properties such as vitamin E, as well as phenolic compounds [5,6]. Thus, almond oil can be considered as a healthy source of fat and can be introduced in the formulation of many foods to increase nutritional properties [7].

Almond oil can be extracted by different ways. Solvent extraction leads to major extraction yields, but oil must be subjected to a refining process prior to consumption, with the consequent loss in many positive attributes. Thus, the preferred system to produce an oil ready for consumption is cold extraction by pressure [8]. Two types of presses can be used. The first is the oil-expeller that needs previous thermal conditioning, leading to an extraction process where temperature may play a significant role and which may be difficult to control due to the changes in the extraction temperature caused by the friction of raw almonds during the extraction [9]. The second type of press that can be used to extract almond oil is the hydraulic press where extraction is completely performed under room temperature and controlled conditions. However, to provide improved sensory characteristics to the oil, a previous roasting treatment can be conducted with almonds. In this case, it is important to adjust the roasting temperature and time to avoid the presence of undesirable compounds derived from too strong of treatments [10].

Almond oil sensory characteristics can be evaluated by sensory tests where judges make an evaluation of properties such as color or taste [11,12]. Almond oil is a relatively new ingredient, and its characteristics are not well-established. In addition, the subjective character of the judges’ evaluations means that the sensory evaluation of almond oil can be biased, leading to unclear classifications. To complement sensory evaluations, the characterization of almond oil can be performed by chemical analysis, but this is an expensive method and does not always offer results useful for determining sensory properties of the oils.

Thus, artificial olfactory systems (or electronic noses, i.e., E-noses), electronic tongues (E-tongues) and computer vision (VxC) can be used as alternatives to traditional test panel systems and chromatographic analysis [13]. These systems are already used successfully in other applications, imitating the biological senses of smell, taste and sight, respectively, while eliminating the components of subjectivity and physical exhaustion [14]. These electrochemical sensors have emerged in recent years as powerful sensory tools that allow for successful qualitative and quantitative evaluation and sensory analysis of basic sensory attributes such as acidity, bitterness, saltiness and sweetness and/or sensory defects produced during the production process. Panagou et al. (2008) [15] highlighted the ability of an electronic nose to differentiate the quality of fermented green table olives based on their volatile profile. Volatile compounds may be the result of many reactions arising from thermal treatments, and contribute to the aroma profile of the products, but some of these compounds—such as heterocyclic amines or varying types of aldehydes—may be harmful to health [16], so it is necessary to determine the point at which they can appear to obtain healthy foods.

Countless applications of artificial olfactory systems have been described in a multitude of industrial sectors [17,18,19,20], since E-noses enable fast and non-destructive measurements with simplicity and low operation costs. In addition, the data obtained from E-nose analysis have shown positive correlations with flavor compounds and sensory notes [21]. However, within the oil sector, studies are mainly focused on olive oil, generally to determine varieties or cultivars or to detect defects such as rancidity [22,23,24,25,26,27], with very few references to other edible oils such as those produced from nuts.

Therefore, the aim of this research is to discriminate almond oils extracted by pressing the roasted almonds at different times and temperatures by using E-nose technology and validating the results obtained with sensory and volatile organic compound profile analyses.

## 2. Materials and Methods

### 2.1. Raw Materials and Oil Extraction

Fresh almonds dried at room temperature from the *Comuna* variety were purchased from local producers. To proceed with roasting treatments, almonds were separated into 5 batches. These batches were subjected to different roasting times (t30, t45, t60 and t90 min), maintaining a roasting temperature of 150 °C in a conventional oven (model 210, J.P. Selecta^®^, Barcelona, Spain). These treatments were repeated 3 times to obtain 3 replicas of each sample. The first batch, used as a control (N), was not subjected to any roasting treatment.

Almond oil was immediately extracted with a hydraulic press (Mecamaq, model DEVF 80, Vila-Sana, Lleida, Spain). Almonds were grinded and subjected to a pressure of 200 bar for 10 min. The resulting oil was centrifuged to remove any solid residue and stored in dark glass bottles under refrigeration conditions until further analysis. The oil extraction yield varied from 35% in natural almonds without roasting treatment to 27% in the oil extracted from the almonds subjected to the highest roasting conditions (150 °C, 90 min) due to a decrease in the moisture caused by the thermal treatment. However, previous studies have shown that the fatty acid profile is not affected by the temperature or the extraction yield [5].

### 2.2. Analyses

#### 2.2.1. Sensory Analysis

Almond oils were evaluated for sensory qualities by a tasting panel located in the CICYTEX (Badajoz, Spain) research center according to the procedure described by Sánchez et al. (2022) [28] and the IOC (2021) [29]. A panel of 10 trained panelists evaluated oils on a structured scale (0 to 10 points) where the points regarding positive and negative odors were annotated. An amount equal to 15 g of almond oil from each treatment was introduced in a standard cup and covered with a watch glass. The samples were placed in a heating block at 28 °C. The positive odors evaluated were related to almond and toasted aromas, and the negative ones, considered as defects, related to burned, dry smells and wood. The results were expressed as average values when the variation coefficient (CV) was less than 20.

#### 2.2.2. Volatile Compound Analysis (VOCs)

VOCs were analyzed using gas chromatography following the method described by López-López et al. (2019) [30] and Montaño et al. (2021) [31]. Aliquots of 2.0 g of oil were placed in a 15 mL glass vial with 7.0 mL of 30% (*w*/*v*) NaCl. The headspace was measured by a StableFlex fiber (polydimethylsiloxane/divinylbenzene, 65 μm, Supelco) at 40 °C for 30 min. The fiber was then inserted into the equipment for its desorption at 250 °C for 15 min. Analyses were performed using a Bruker Scion 456-GC triple quadrupole gas chromatograph with a DB WAXETR capillary column (60 m × 0.25 mm; ID: 0.25 mm) [28]. The identification of the compound was based on the coincidence of mass spectra with the NIST standard reference database.

#### 2.2.3. E-Nose

The E-nose used was designed by the University of Extremadura (Spain) [32]. It consists of an array of 11 latest-generation metal oxide (MOX) sensors spread over four chips: BME680 from Bosch, SGP30 from Sensirion and CCS811 and iAQ-Core from ScioSense [33]. The sensors of the portable E-nose (39 mm × 33 mm) measure the headspace of the samples and send data to a smartphone via Bluetooth. The E-nose measurement was conducted at the same time as the sensory panel. The E-nose data collection was divided into two phases to ensure the correct response of the sensors to each sample: (i) adsorption phase, where the volatile compounds of the samples were put in contact with the sensors for 60 s; and (ii) desorption phase, where sensors were put in contact with the air in an empty cup for 30 s to obtain the baseline. The E-nose recorded data at one-second intervals, and the system took a reading of the resistive value supplied by each sensor. Eight measurements were taken for each sample of almond oils. The data obtained with this equipment were processed to obtain a response from each of the sensors to the sample by calculating the maximum signal value minus the minimum signal value multiplied by 100 and subtracted by one. After that, multivariate analysis was performed.

### 2.3. Statistical Analysis

ANOVA followed by Tukey’s test were performed to establish statistically significant differences between the sensory properties of the different almond oils (*p* < 0.05). SPSS 18.0 software was used for statistical analysis (SPSS Inc., Chicago, IL, USA). Data were expressed as means and standard deviations (SD).

The data obtained by the E-nose were processed by MATLAB software. These data were treated by an unsupervised exploratory principal component analysis (PCA) [34]. This analysis was used to show how the values of the almond oils were grouped according to their volatile compound profile. By performing a PCA, a reduction in the dimension of the input variables could be carried out, thereby obtaining principal components that were linear combinations of original response vectors. Since the study variables were measured according to different units, the original variables were auto-scaled.

Next, a supervised classification analysis in the form of a partial least squares discriminant analysis (PLS-DA) was applied [35] to build a model to discriminate between almond oils produced by almonds subjected to different thermal treatments. A confusion matrix was constructed to derive the cross-validation predictions. Further, the partial least squares (PLS) method was also used to build quantification models for the evaluation of a burned aroma defect perceived by the panelist. The chemometric method was described by Sánchez et al. (2022) [19]. Almond oils were divided into a calibration set that contained 70% of all samples and a validation set that contained the remaining samples (30%). Samples were divided randomly between the two sets. Data analysis was performed using MATLAB version R2016b, version 9.1 (The Mathworks Inc., Natick, MA, USA) with PLS_Toolbox 8.2.1 (Eigenvector Research Inc., Wenatchee, WA, USA).

## 3. Results and Discussion

To evaluate the effectiveness of the electronic nose in relation to the detection of possible defects or differences in the aromatic characteristics of the different almond oils analyzed, they were first analyzed from a sensory point of view by a trained panel and from a chemical point of view by analysis with gas chromatography; then, the data were compared with those obtained by applying the E-nose.

### 3.1. Sensory Aroma of Almond Oils

The almond oils obtained from roasted almonds were sensorily evaluated by a tasting panel to classify them according to the predominantly perceived defect (PPD) (Table 1). Some significant differences between the different almond oils were obtained for each sensory attribute. Natural oil (N), extracted from almonds that were not subjected to thermal treatments, only showed positive attributes, with a low-intensity almond odor. In this sample, tasters did not detect any negative attributes. When the oil was obtained from almonds subjected to a light roasting treatment (t30), the intensity of the positive attributes (almond and toasted odor) was the highest of all samples. Similar to the natural sample, no defects were found in t30.

However, when almond kernels were subjected to more intense roasting treatments, some sensory defects associated with a burned odor were detected in the extracted oils. The main defects found were burned and dry smells from 3.9 to 7.2 points, although the values of the burned attribute were greater than the other. Another negative attribute was the smell of wood, which was only detected in the oil extracted from the almonds subjected to the most intense thermal treatment. The highest thermal treatments (t90) provoked the highest score for these attributes, while the lowest was found in t45.

On the other hand, the thermal treatment applied to the almond seeds before oil extraction influenced the intensity of the positive sensory attributes of the almond oils detected by the tasters (almond and toasted odors). Thus, it was observed that the almond oils showed lower values of the positive attribute when the intensity of the thermal treatment increased. In fact, the higher roasting treatments caused the disappearance of these positive attributes.

Taking into account only sensory attributes, and considering the classification made for the most commonly used olive oil [36], N and t30 were the only treatments that would be classified as the ‘extra virgin’ category, because only positive attributes were detected in the extracted oils. T45 treatment could be classified as ‘virgin oil’ because the intensity of the major defects were less than 3.5 points. T60 and t90 would be classified as ‘lampante oils’ because the intensity of the majority defect was greater than 3.5 points. Thus, only the first three experimental treatments could be legally marketed when considering significant defects. This result has interesting consequences for nut oil producers who are required to carry out necessary thermal treatments to achieve a desirable odor quality during the elaboration process.

### 3.2. Volatile Compounds of Almond Oils

**Figure 1:**
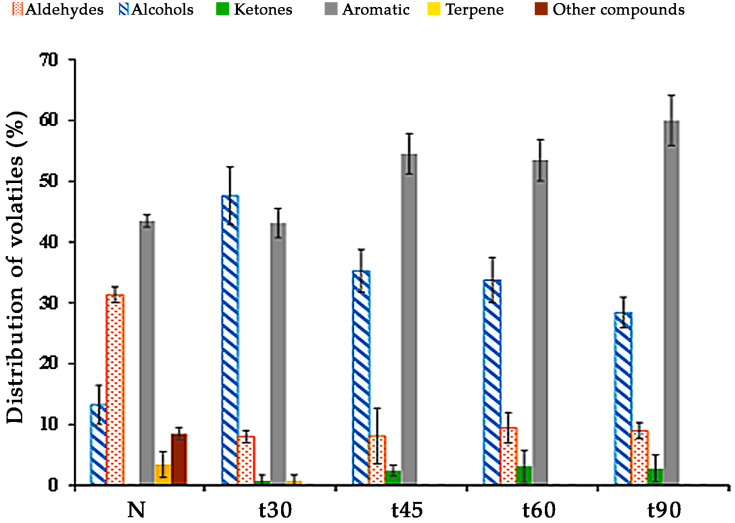
Chemical distribution of volatile compounds of oils from almonds subjected to different thermal treatments before oil extraction.

Figure 1 shows the distribution of volatile compounds classified according to different families of almond oils in which kernels were subjected to different thermal treatments. There are statistically significant differences between the different families of volatile compounds analyzed in the studied almond oils. Most of the volatile compounds were aldehydes, aromatics and alcohols, while the minor ones were terpenes and other compounds. Some of the families studied increased or decreased depending on the application of the roasting treatments to the almonds before the oil-extraction process.

Aldehydes increased significantly for the different studied almond oils. The lowest contents were obtained from the naturally extracted oils (N). Almond seeds with different thermal treatments presented 53–72% more of these compounds than oil from natural almonds (N). Alcohols increased significantly in N in relation to almond oils from roasted almonds (70%). Ketones increased with the increase in the thermal treatments, and were not present in N. The concentration of aromatic compounds was similar between N and t30; however, this content increased significantly in the most intense thermal treatments (t45–t90). Terpenes were only present in the N and t30 treatments, and the other compounds were only found in N.

Table 2 shows the volatile compound profiles of almond oils made from seeds which were subjected to different thermal treatments. In total, 40 volatile organic compounds (VOCs) were identified that were distributed in the families shown in Figure 1 according to their chemical nature. The main VOCs were 2-methyl-butanal, hexanal, 2,3-butanediol, toluene, 2,5-dimethyl-pyrazine and 2-ethyl-5-methyl-pyrazine.

The compounds included in the aldehyde and aromatic families were the most abundant among the VOCs detected. In previous studies, it has been observed that roasting enhanced the concentration of aldehydes formed by Strecker degradation of valine, leucine, isoleucine and phenylalanine, generating compounds such as 2- and 3-methylbutanal [37]. The results showed that higher amounts of some aldehydes were found in the studied almond oils. Hexanal was the only one that presented the highest amount in N and the lowest in oils from roasted almonds. The odor of this compound was related to a fruity characteristic. The other volatile compounds within the aldehyde family were found in greater concentrations in the oils from almonds subjected to the softer thermal treatment (t30), decreasing with an increasing roasting time. The exception to this behavior was found in 3-furaldehyde and benzaldehyde, whose concentration increased in oils from almonds subjected to long roasting times. These compounds could be related to unpleasant odors, which would agree with the results obtained from the sensory analysis. In this sense, benzaldehyde is related to the characteristic odor of bitter almond, which increases as the roasting time lengthens [38].

All the alcoholic compounds decreased their content with the application of the thermal treatments studied. The main volatile compounds were 3-methyl-1-butanol, 2-methyl-1-butanol and 2,3-butanediol, related to aromas such as pungent, fruity, sweet and alcoholic.

Other volatiles from the aromatic family produced unpleasant odors such as 4-methyl-pyrimidine, 2,5-dimethyl-pyrazine, 2-ethyl-6-methyl-pyrazine, 2-ethyl-5-methyl-pyrazine and pyrazinamide, which give sensory properties to the oils relating to roasted, butter, burnt, smoky, musty or woody aroma. On the other hand, toluene was found in higher concentrations in N and was not present in the rest of the studied almond oils. This compound is related to fruity and sweet aromas.

### 3.3. Discrimination of Almond Oils with E-Nose

**Figure 2:**
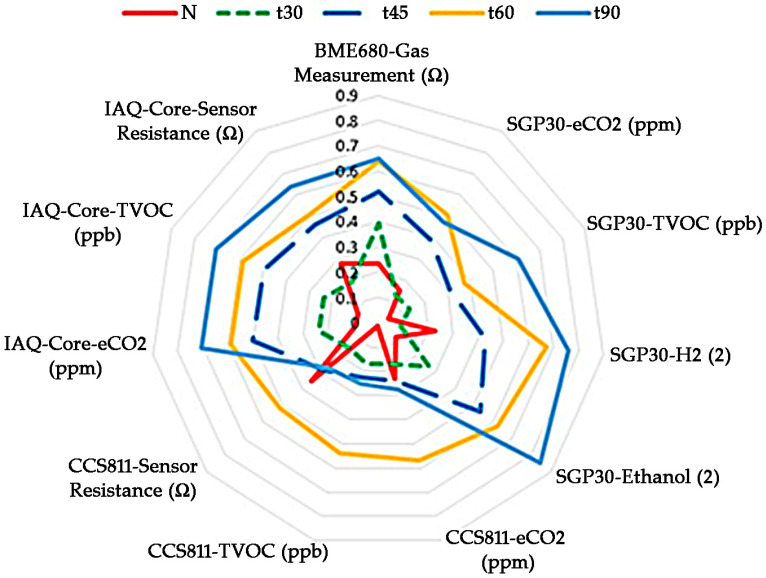
Radial plots of the sensors’ responses to different thermal treatments applied to fresh almonds before oil extraction.

The data obtained with the E-nose were previously processed to characterize the response of each of the sensors to the olfactory profile of each of the samples. For this, a radial diagram was made (Figure 2). To auto-scale the data obtained by samples submitted to roasted treatments, the following formula was applied: (Xi − XMIN)/(XMAX − XMIN); Xi: experimental value for sample i; XMIN: minimum experimental value of the data series; and XMAX: maximum experimental value of the data series. Data obtained was represented in the radial graph. As observed in the figure, each sample presented a different signal response which could be related to the different aroma of the samples studied. Furthermore, the responses of all sensors to each samples studied were completely different. The amplitude of the signals increased with the increase in the thermal treatments applied to fresh almonds.

**Figure 3:**
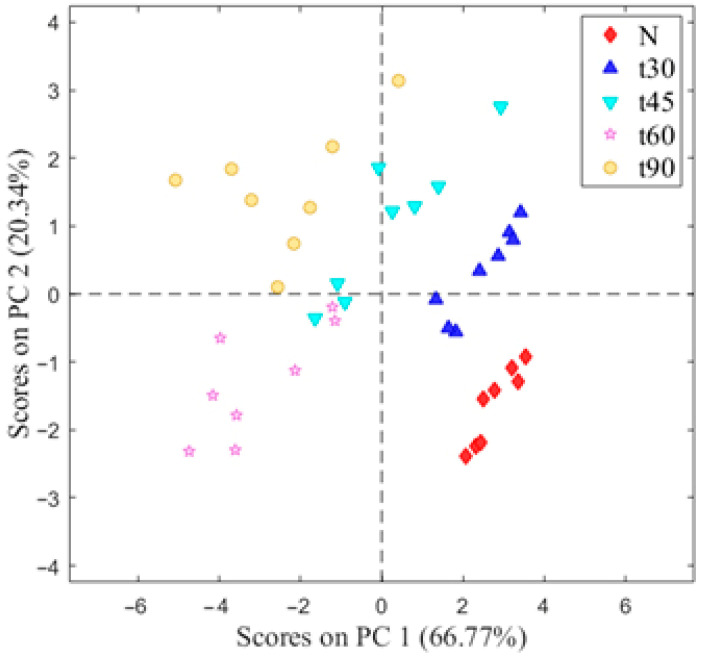
Score plot of the PCA analysis for oils extracted from almonds subjected to different thermal treatments before oil extraction.

The E-nose data from the studied almond oils showed multiple variables. To determine the relationships between the E-nose-measured variables, the first step was to reduce the variables using a principal component analysis (PCA). The PCA plots visually represent how similar data are grouped together. Figure 3 shows a first separation between N and almond oils from kernels subjected to different thermal treatments.

The PCA results showed that 66.7% of the total variance of data was explained by PC1 and 20.3% by PC2. This model based on the first two components showed a clear differentiation of the samples according to the volatile compounds profile. In this sense, almond oils from kernels subjected to different thermal treatments were separated in different groups. In addition, the oil extracted from untreated almonds (N) also clearly differed from the rest.

A classification analysis was then performed using the PLS-DA and leave-one-out cross-validation. Thus, the classification results as a confusion matrix are shown in Table 3. The sum of the diagonal elements of the confusion matrix gives the percentage of success in the classification; in this case, 87.5% was obtained.

These results prove the ability and accuracy of the E-nose to discriminate between different almond oils which seeds were thermally treated. Thus, the outcomes show that the E-nose is able to discriminate between oils according to their aroma. These findings are consistent with those obtained by the sensory (Table 1) and volatile profile (Table 2) analyses, differentiating between almond oils and confirming the suitability of the E-nose for distinguishing between different thermal treatments. This is an interesting result, given that this tool could be useful at the industrial level in detecting positive and negative attributes after the elaboration process. Therefore, oil quality could be increased in this sector.

**Figure 4:**
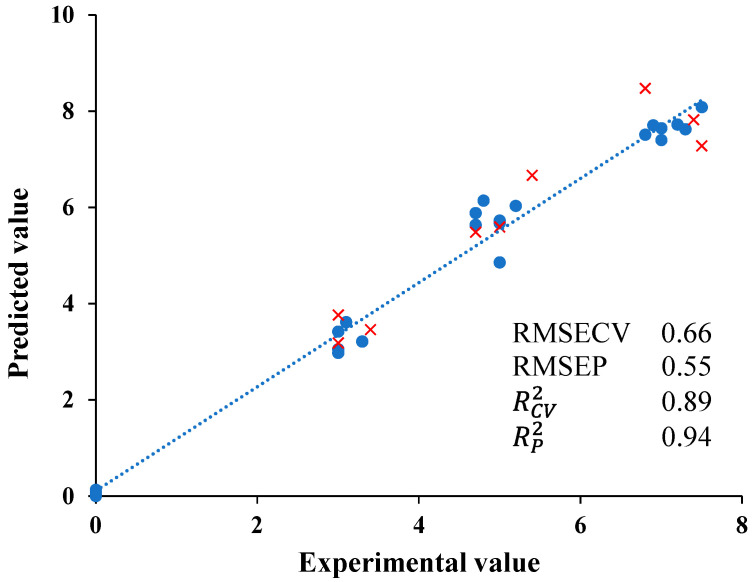
Experimental values against PLS cross-validation predictions (●) and validation set predictions (×) for burned aroma defect perceived by panelists.

Finally, with the aim of assessing the use of E-nose to quantify the quality of almond oils, a partial least squares (PLS) chemometric approach was taken (Figure 4). PLS regression was used to establish prediction models from the burned defect perceived by the tasting panel and data produced by E-nose signals. $R_{CV}^{2}$ values for the models established for a burned defect were 0.89. The root-mean-standard error for cross-validation (RMSECV) and the root-mean-standard error for prediction validation (RMSEP) were also calculated. Low RMSECV values of 0.66 were also estimated. The PLS calibration model was validated using samples that were not included in the calibration test. The validation parameters obtained were also acceptable. $R_{P}^{2}$ values were 0.94 for negative aroma perceived whilst RMSEP values were 0.55. Thus, this model allows for the quantification of the burned defect of samples in order to observe possible alterations to the almond oil once it has been elaborated and to classify them according to their quality. The bibliography indicates some results related to the discrimination and quantification of certain parameters in foods with electronic devices. Rodríguez et al. (2009) [39] were also able to classify toasted coffee samples with different qualities by E-nose in order to observe the excellence of Colombian coffee. Sánchez et al. (2022) [28] established a chemometric model to classify Californian-style table olives according to the cooking effect provoked by thermal sterilization.

## 4. Conclusions

When almond oil is extracted, a prior roasting treatment applied to the almond kernels may help increase positive sensory attributes such as almond odor and toasting odor in the oil. However, when thermal treatment is too intense, the positive sensory attributes decrease, and negative attributes such as a burned, dry smell or woodiness appear. Thus, the thermal treatment must be carefully controlled to obtain extra virgin almond oil without sensory defects.

In a similar way, the thermal treatment of almonds causes a change in the content of volatile compounds measured in the oils. The content of aldehydes and aromatic compounds increases in oils from roasted almonds, while alcohols and terpenes decrease. Furthermore, in natural almond oils, compounds associated with fruity aromas such as hexanal or toluene showed the highest values while aromatic compounds such as 4-methyl-pyrimidine, 2,5-dimethyl-pyrazine, 2-ethyl-6-methyl-pyrazine, 2-ethyl-5-methyl-pyrazine and pyrazinamide—related to roasted, butter, burned, smoky, musty or woody aromas—appeared only when the almonds were subjected to thermal treatments. These data are in agreement with sensory analysis.

Finally, the E-nose proved to be a powerful tool with an analytical capacity to discriminate between VOCs derived from almond oils. The classification provided with this device coincides with the results provided by the tasting panel and with the VOC profile. Further, results obtained by the PLS models suggest that the electronic device may be used to estimate the burned defect perceived by the tasting panel with the advantage of being an objective model in order to classify almond oils according to their quality by using only an E-nose. Thus, the electronic device was able to detect the olfactory pattern of the samples with different qualities. This proves that—combined with chemometric tools, and as an auxiliary tool in the tasting panel—the E-nose represents a fast, simple, reliable and low-cost method suitable for use at industrial level to control the quality of this product. However, further testing may be needed to validate the effectiveness of the system with commercial oils.

## Figures and Tables

**Table 1:** Predominantly perceived sensory defects (mean ± standard deviation) of oils from almond seeds subjected to different thermal treatments before oil extraction. Different lowercase letters indicate significant differences between thermal treatments, while capital letters show significant differences between attributes (Tukey’s test, *p* < 0.05).

	N	t30	t45	t60	t90
**Positive attribute**					
Almond	1.1 ± 0.1 c	6.1 ± 0.3 aA	4.3 ± 0.2 bA	4.5 ± 0.3 bA	n.d
Toasted	n.d	4.0 ± 0.3 aB	2.3 ± 0.3 bB	1.5 ± 0.3 bB	n.d
**Negative attribute**					
Burned	n.d	n.d	3.1 ± 0.4 bA	4.8 ± 0.3 bA	7.2 ± 0.5 aA
Dry smell	n.d	n.d	2.9 ± 0.3 nsA	3.9± 0.4 nsA	4.8 ± 0.2 nsB
Wood	n.d	n.d	n.d	n.d	3.1 ± 0.3

N: natural oil without thermal treatment; t30: almond seeds subjected to 30 min of thermal treatment before oil extraction; t45: almond seeds subjected to 45 min of thermal treatment before oil extraction; t60: almond seeds subjected to 60 min of thermal treatment before oil extraction; t90: almond seeds subjected to 90 min of thermal treatment before oil extraction. Thermal treatment: 150 °C in all cases. n.d: not detected.

**Table 2:** Content of volatile compounds (mean %, n = 3) obtained from oils extracted from almond seeds subjected to different thermal treatments before oil extraction. R.T.: retention time.

Volatile Compounds	R.T. (min)	N	t30	t45	t60	t90
**Aldehydes**						
3-methyl-butanal	2.8	n.d.	10.6	4.7	5.2	1.9
2-methyl-butanal	3.0	n.d.	17.6	14.4	11.0	8.9
Hexanal	6.7	13.2	3.4	2.7	1.9	1.4
3-Furaldehyde	8.2	n.d.	4.0	8.8	9.1	9.4
Benzaldehyde	14.8	n.d.	0.7	2.2	3.4	4.0
Benzene acetaldehyde	19.2	n.d.	11.3	2.5	3.2	2.8
**Alcohols**						
dimethyl-silanediol	3.6	6.6	n.d.	n.d.	n.d.	n.d.
3-methyl-1-butanol	4.4	5.0	1.5	1.0	1.0	1.0
2-methyl-1-butanol	4.5	4.4	n.d.	n.d.	n.d.	n.d.
1-Pentanol	5.5	n.d.	0.7	0.9	0.8	1.0
2,3-Butanediol	6.2	4.8	2.6	0.6	1.0	0.6
(S)-2-Methyl-1-butanol	4.5	4.4	n.d.	n.d.	n.d.	n.d.
3-Furanmethanol	9.4	n.d.	0.0	3.1	4.2	5.3
2-Furanmethanol	9.4	n.d.	1.6	1.0	1.0	0.0
Eucalyptol	18.3	0.5	n.d.	n.d.	n.d.	n.d.
1-Hexanol	10.1	5.6	1.6	1.5	1.5	1.0
**Ketones**						
Acetoin	3.7	n.d.	0.7	0.2	n.d.	n.d.
3-hydroxy-2-butanone	3.7	n.d.	n.d.	n.d.	0.3	0.2
2-hydroxy-3-methyl-2-cyclopenten-1-one	18.2	n.d.	n.d.	1.2	1.3	1.2
2,3-Butanedione	10.0	0.0	0.0	1.0	1.5	1.4
**Aromatic**						
1-methyl-1H-pyrrole	4.4	n.d.	n.d.	0.7	0.6	0.4
Pyrrole	5.0	n.d.	1.6	3.8	3.3	3.5
Toluene	5.3	38.8	1.1	1.0	1.0	1.0
Dihydro-2-methyl-3(2H)-Furanone	7.0	n.d.	n.d.	1.2	1.4	1.3
4-methyl-pyrimidine	7.7	n.d.	6.3	10.1	8.9	10.3
Ethylbenzene	9.3	0.5	n.d.	n.d.	n.d.	n.d.
p-Xylene	9.8	1.6	0.8	n.d.	n.d.	n.d.
o-Xylene	9.8	1.9	n.d.	n.d.	n.d.	n.d.
1-Pyrrolidinebutyronitrile	10.7	n.d.	n.d.	1.0	1.1	1.2
2,5-dimethyl-pyrazine	12.1	n.d.	12.5	15.9	16.4	20.6
ethyl-pyrazine	12.2	n.d.	8.2	n.d.	n.d.	n.d.
1-ethyl-2-methyl-benzene	14.6	0.7	n.d.	n.d.	n.d.	n.d.
4H-Pyran-4-one, 2,3-dihydro-3,5-dihydroxy-6-methyl-/Cyclotetrasiloxane, octamethyl-	15.8	n.d.	1.5	1.6	1.3	1.6
2-ethyl-6-methyl-pyrazine	16.6	n.d.	1.4	4.3	6.9	7.5
2-ethyl-5-methyl-pyrazine	16.9	n.d.	9.7	12.4	10.4	9.4
Pyrazinamide	17.9	n.d.	n.d.	2.5	2.3	3.1
**Terpene**						
trans-.beta-Ocimene	13.0	1.4	n.d.	n.d.	n.d.	n.d.
alpha-Pinene	17.0	1.4	n.d.	n.d.	n.d.	n.d.
Limonene	18.2	0.7	0.7	n.d.	n.d.	n.d.
**Other compounds**						
octamethyl-cyclotetrasiloxane	15.8	8.5	n.d.	n.d.	n.d.	n.d.

N: natural oil without thermal treatment; t30: almond seeds subjected to 30 min of thermal treatment before oil extraction; t45: almond seeds subjected to 45 min of thermal treatment before oil extraction; t60: almond seeds subjected to 60 min of thermal treatment before oil extraction; t90: almond seeds subjected to 90 min of thermal treatment before oil extraction. Thermal treatment: 150 °C in all cases. n.d: not detected.

**Table 3:** Confusion matrix obtained through PLS-DA for discrimination between almond seeds subjected to different thermal treatments before oil extraction. Values are expressed in percentage.

Predicted Class					
Real Class	N	t30	t45	t60	t90
N	20.0	0	0	0	0
t30	0	20.0	0	0	0
t45	0	0	17.5	0	2.5
t60	0	0	2.5	15.0	2.5
t90	0	0	0	5.0	15.0

N: natural oil without thermal treatment; t30: almond seeds subjected to 30 min of thermal treatment before oil extraction; t45: almond seeds subjected to 45 min of thermal treatment before oil extraction; t60: almond seeds subjected to 60 min of thermal treatment before oil extraction; t90: almond seeds subjected to 90 min of thermal treatment before oil extraction. Thermal treatment: 150 °C in all cases.

## Data Availability

Not applicable.
